# Decoding the skin microbiota: A Mendelian randomization study reveals new insights into acne causality

**DOI:** 10.1097/MD.0000000000042865

**Published:** 2025-06-13

**Authors:** Jie Zhou, Yixin Xu, Haitao Wang

**Affiliations:** a Department of General Surgery, The Wujin Hospital Affiliated with Jiangsu University, Changzhou, China; b Department of General Surgery, The Wujin Clinical College of Xuzhou Medical University, Changzhou, China; c Department of General Surgery, The Third Affiliated Hospital of Soochow University, Changzhou, China.

**Keywords:** causal relationship, genetically predicted, GWAS, microorganism, single nucleotide polymorphism

## Abstract

The link between skin microbiota and acne, despite being a research focus for years, still lacks full understanding and remains controversial. The genome-wide association studies data utilized in this study were all sourced from public databases. Microbiota data from sebaceous (acne-prone) skin regions were selected for bidirectional Mendelian randomization analysis with acne. The primary method utilized was the inverse-variance weighted (IVW) method, supported by heterogeneity analysis, horizontal pleiotropy testing, outlier detection, and “leave-one-out” sensitivity analysis. This Mendelian randomization analysis revealed causal connections between the abundance of the genus: staphylococcus (IVW odds ratio [OR]: 1.074; 95% confidence interval [CI]: 1.031–1.119, *P* = .001) and Propionibacterium acnes (IVW OR *=* 1.039, 95% CI *=* 1.007–1.073, *P* = .017) in sebaceous skin and the risk of acne development. Additionally, ASV004 (Corynebacterium [unclassified]) (IVW OR *=* 0.967, 95% CI *=* 0.937–0.999, *P* = .044) and ASV008 (Staphylococcus [unclassified]) (IVW OR *=* 0.943, 95% CI *=* 0.897–0.991, *P* = .021) were identified as protective factors that could reduce acne incidence. No reverse causality between skin microbiota and acne was found, with all analyses showing no horizontal pleiotropy or heterogeneity. This study is instrumental in exploring the link between skin microbiota and acne, supporting theoretical debates, and shedding light on acne’s pathogenesis.

## 1. Introduction

Acne is a prevalent chronic skin condition that primarily originates in the hair follicles and is often accompanied by inflammation. It is estimated that about 85% of adolescents and young adults are affected by acne,^[1]^ with moderate to severe cases comprising approximately 15% to 20% of these instances.^[2]^ According to the Global Burden of Disease Study in 2013, acne accounts for 0.29% of all skin diseases and 1.79% of the global disease burden, making it the second most common skin condition, following only dermatitis.^[3]^

Currently, the exact causes of acne are not fully understood. Factors such as excessive sebum production, abnormal proliferation and differentiation of keratin-forming cells within hair follicles, bacterial colonization, and host inflammatory responses are believed to play a role.^[4]^ Extensive research indicates that the genetic architecture of acne is complex, involving multiple susceptibility loci. Understanding the multifactorial nature of acne’s pathogenesis is crucial for its prevention, treatment, and management.^[5,6]^

The skin is home to hundreds of microbial species, which form various communities based on different skin environmental niches.^[7]^ When the balance of these normal microbial communities is disrupted, or the host’s immune defense capabilities are diminished, opportunistic microbes may trigger or exacerbate certain skin diseases.^[8]^ For a long time, the relationship between skin microbiota and acne has been a focal point of research, yet this relationship has not been fully elucidated.^[9]^ For instance, Propionibacterium acnes (P. acnes), the most common and abundant species found within the sebaceous follicles where acne develops, accounts for approximately 90% of the skin’s microbiome.^[10,11]^ It is believed to play a role in acne’s progression through several mechanisms, including modulating the activity of sebaceous glands,^[12]^ encouraging the formation of comedones,^[13]^ and initiating inflammatory responses in the host.^[14]^ Moreover, research has revealed that P. acnes prevails in the follicular sebaceous gland microbiota of both acne patients and individuals with clear skin, showing comparable quantities or relative abundances between the 2 groups, sometimes even slightly higher in those with healthy skin.^[10,11]^ Concurrently, P. acnes is recognized as a crucial symbiotic bacterium for skin health. By decomposing triglycerides to release free fatty acids, it helps maintain a low pH on the skin surface, which in turn inhibits the growth of pathogenic bacteria such as Staphylococcus aureus and Streptococcus species^[15,16]^ Thus, the exact role of P. acnes in acne’s pathogenesis remains to be further elucidated through comprehensive research.^[9]^ Furthermore, alongside P. acne, a multitude of additional bacterial species make their home on the skin surface, some of which might contribute to either the preservation of skin health or the advancement of disease states. For instance, alterations in the presence of Staphylococcus epidermidis, Staphylococcus hominis, and other coagulase-negative staphylococci have been observed on the skin of individuals with acne.^[17–19]^ Hence, the intricate interplay between host and pathogen within the skin ecosystem often manifests through straightforward mappings and recognizable inflammatory profiles. These observations underscore the practicality of simplistic models, while simultaneously suggesting their inherent limitations.

Currently, insights into the causal relationship between skin microbiota and acne largely stem from observational studies, which come with inherent limitations. These limitations encompass an incomplete identification of the skin microbiota involved, confounding factors that are either not measured or inaccurately measured, the possibility of reverse causation, and various other sources of bias. In an effort to navigate past these constraints, employing Mendelian randomization (MR) analysis with data from genome-wide association studies (GWAS) has become a promising strategy to investigate causal relationships within assumed exposure-outcome pathways.^[20]^ MR leverages the principle of random allocation of genetic variants at conception, effectively simulating a natural experiment. This method facilitates the exploration of potential causal links between risk factors (skin microbiota) and disease outcomes (acne), ensuring that the influence of confounding variables is minimized through their random distribution.^[21]^ In our study, we’ve meticulously gathered the most up-to-date GWAS summaries focusing on the skin microbiota of the forehead, a region notable for its higher sebum production and susceptibility to acne. To our knowledge, no studies have yet employed a 2-sample MR analysis to explore in depth the complex interactions between the unique microbiome of high sebum secretion areas and the occurrence of acne. This is crucial for unraveling the role of the skin microbiota in acne and identifying future therapeutic targets.

## 2. Methods

### 2.1. Study design

In this study, all data were obtained from publicly available databases and received approval from the relevant research institution’s review board, thereby bypassing the need for ethical committee review.

Our research employed single nucleotide polymorphisms (SNPs) as instrumental variables (IVs) to conduct a comprehensive bidirectional MR analysis, investigating the causal relationship between human skin microbiota and acne.^[22]^ Additionally, MR analysis must adhere to 3 critical assumptions: SNPs are associated with the exposure; SNPs are independent of confounding factors in the exposure-outcome relationship; SNPs solely influence the outcome through the exposure.^[23]^

### 2.2. GWAS summary data sources

#### 2.2.1. *Data for human skin microbiota*:

The GWAS data utilized in this study on the human skin microbiota originated from 2 population-based, cross-sectional German cohorts, namely KORA FF4 (comprising 324 participants) and PopGen (comprising 273 participants), totaling 1656 skin samples.^[24]^ These samples were obtained from various skin environments: dry (dorsal and volar forearm [PopGen]), moist (antecubital fossa [KORA FF4 and PopGen]) and sebaceous (retroauricular fold [KORA FF4] and forehead [PopGen]) (Table S1, Supplemental Digital Content, https://links.lww.com/MD/P191). Genome-wide association analyses were conducted on univariate relative abundances of individual bacteria (amplicon sequence variants; ASVs) and nonredundant taxonomic groups ranging from genus to phylum levels. The microbial community characteristics within these skin samples were determined through sequencing the V1-V2 region of the 16S ribosomal RNA gene. The GWAS summary statistics for the 150 human skin microbiota can be accessed in the GWAS catalog, available under the codes GCST90133164 to GCST90133313 (Table S1, Supplemental Digital Content, https://links.lww.com/MD/P191). The skin microbiota can vary significantly across different body sites and is closely related to the occurrence and development of various diseases. Given that acne primarily develops in areas with dense sebaceous glands, this study will specifically analyze the relationship between 23 types of microbiota on sebaceous skin and acne (Table 1).

**Table 1:** Details of the GWAS and datasets used in our analyses.

Phenotypes	Cases/controls	Consortium/author	Population	PubMed ID	Data download link
Human Skin Microbiota	KORA FF4 (n = 324)	Lucas Moitinho-Silva et al	German	36261456	https://www.ebi.ac.uk/gwas/; Accession numbers GCST90133164–GCST90133313
	PopGen (n = 273)				
Acne	3245/394,105	FinnGen consortium	European	–	https://storage.googleapis.com/finngen-public-data-r10/summary_stats/finngen_R10_L12_ACNE.gz

GWAS = genome-wide association studies.

#### 2.2.2. Data for acne:

The latest summary-level statistics regarding acne were derived from the FinnGen project’s GWAS data (Release 10). This dataset includes information from 3245 individuals diagnosed with acne and 394,105 control subjects. The specific phenotype associated with this data is denoted as “L12_ACNE.” For further analysis and research purposes, the dataset is publicly accessible via the following link: https://storage.googleapis.com/finngen-public-data-r10/summary_stats/finngen_R10_L12_ACNE.gz (Table 1).

### 2.3. IVs selection and data harmonization

In our investigation, we adhered to rigorous selection criteria for SNPs to bolster the robustness of our findings. In MR analysis, the standard approach is to select SNPs that reach genome-wide significance (*P <* 5 × 10^−8^) for analysis. However, when strictly applying this criterion, many skin microbiota lack sufficient IVs for analysis. To ensure an ample supply of IVs, we adhered to the research protocol put forth in the original study,^[24]^ selecting SNPs that reached genome-wide significance (*P <* 1 × 10^−5^) for further meticulous examination. Palindromic and ambiguous SNPs were meticulously excluded from our analysis to maintain the integrity of our IVs.^[25]^ We then grouped SNPs based on linkage disequilibrium, utilizing a 10,000 kb window and an *r*^2^ threshold under 0.001. The *F*-statistic, calculated using the formula [(N − *K* − 1)/*K*]/[*R*^2^/(1 − *R*^2^)], served as a quantitative measure of the variance each SNP accounted for, where *K* is the count of genetic instruments and N the sample size. The *F*-statistic is commonly used to evaluate the strength of an IV by measuring its correlation with the exposure. An *F*-statistic > 10 is typically considered indicative of a strong IV, reflecting sufficient correlation. To ensure the robustness and reliability of our analysis, we excluded IVs with an *F*-statistic < 10.^[26]^ Lastly, our extensive literature review aimed to rigorously assess all phenotypes associated with the genetic instruments employed in our study, and we carefully eliminated SNPs potentially entangled with confounding variables to ensure the validity of our causal inferences.

### 2.4. Statistical analysis

For our thorough and comprehensive analysis, we used R software (version 4.2.0, https://www.r-project.org) along with the “Two-Sample MR” package (version 0.5.6) to conduct MR analysis.^[27]^

### 2.5. Primary analysis

To explore the causal relationship between 23 types of sebaceous skin microbiota and acne, we conducted a 2-sample MR analysis. First, we used GWAS data for these microbiota as exposure and GWAS data for acne as the outcome. Notably, the inverse-variance weighted (IVW) method is the most important analytical approach in this study. It combines meta-analysis strategies with the Wald estimates for each SNP. In the absence of horizontal pleiotropy, the IVW results are unbiased.^[28]^ Results are considered significant when *P* *<* .05. Additionally, we employed supplementary methods such as Bayesian weighted MR (BWMR)^[29]^ and the weighted median method.^[30]^ Specifically, the BWMR method is designed for causal inference and can address uncertainties caused by weak effects in polygenic traits. This method uses Bayesian weighting to identify outliers and handle violations of the IV assumptions due to pleiotropy. Compared to the MR-Egger method, the weighted median method has a smaller standard deviation, thus providing higher precision. The weighted median method can yield consistent estimates even in the presence of horizontal pleiotropy, treating up to 50% of genetic variations as invalid instruments.^[31]^

### 2.6. Reverse MR analysis

To explore whether there is a potential reverse causal relationship between these 23 sebaceous skin microbiota and acne, we conducted a reverse MR analysis with acne as the exposure variable and skin microbiota as the outcome variable. The methods and standards for the reverse MR analysis were consistent with those described above.

### 2.7. Sensitivity analysis

We employed several methods to enhance the reliability of our conclusions. First, in the 2-sample MR analysis, we considered potential heterogeneity due to differences in experimental conditions, study populations, and SNPs, which could affect the estimation of causal effects. To address this, we used the IVW and MR-Egger methods to assess heterogeneity. We evaluated the heterogeneity of the genetic instruments using Cochrane *Q* statistic, with a *P*-value > .05 indicating nonsignificant heterogeneity.^[32]^ Second, a fundamental assumption in MR analysis is that the IV affects the outcome only through the exposure, making it necessary to examine potential horizontal pleiotropy between the exposure and the outcome.^[33]^ We used the MR-Egger intercept method to assess the presence of pleiotropy, with a *P*-value >.05 suggesting that pleiotropy is minimal or negligible, thereby excluding its influence on the causal analysis. Additionally, we identified and excluded outliers in the IVW analysis using the MR-PRESSO test.^[34]^ Finally, we conducted a “leave-one-out” analysis to determine the genetic causal effect of individual SNPs on the exposure-outcome relationship.^[35]^

## 3. Results

**Figure 1.**
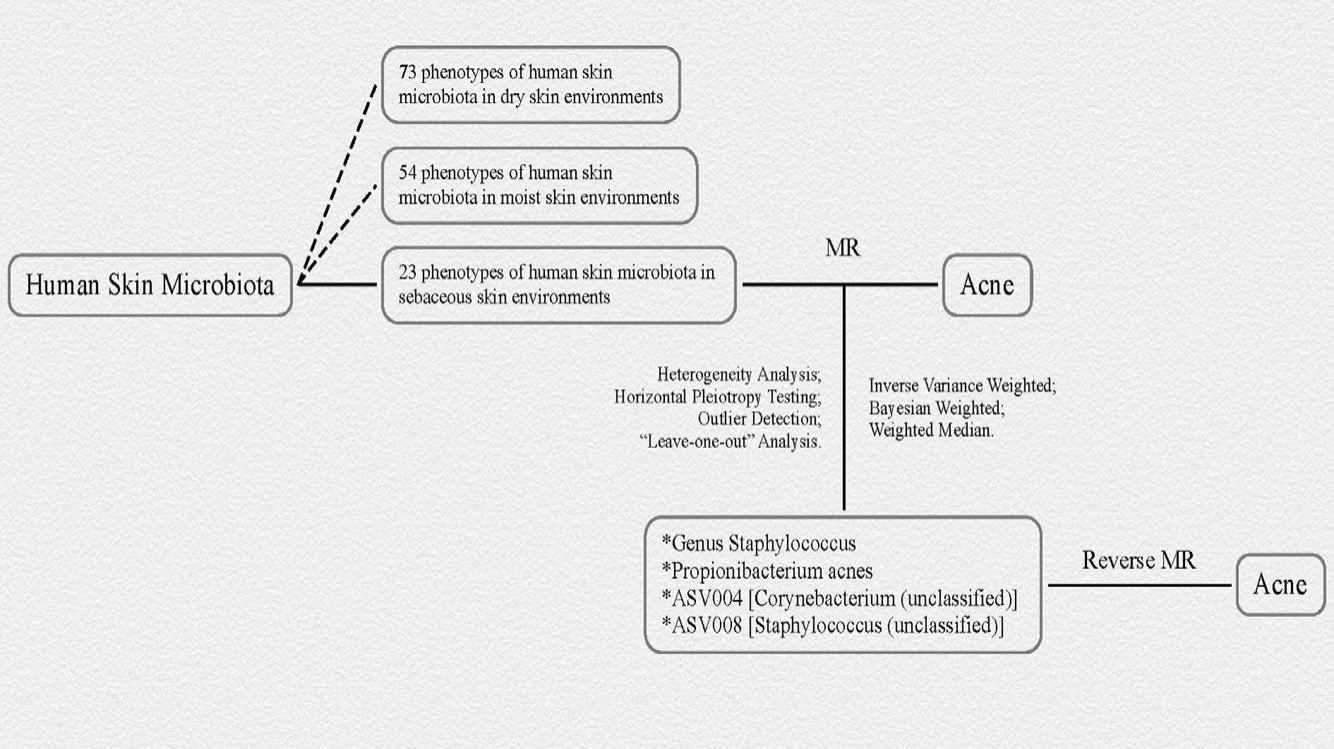
The schematic representation of the study design. ASV = amplicon sequence variant, MR = Mendelian randomization.

Refer to Figure 1 for a detailed schematic diagram of the study design.

### 3.1. Association of 23 phenotypes of sebaceous skin microbiota and acne

To explore the causal relationship between skin microbiota and acne, we selected 23 types of sebaceous skin microbiota from 150 identified skin microbes as exposure variables. We then conducted a systematic 2-sample MR analysis using GWAS data for acne from the FinnGen project as the outcome.

**Figure 2.**
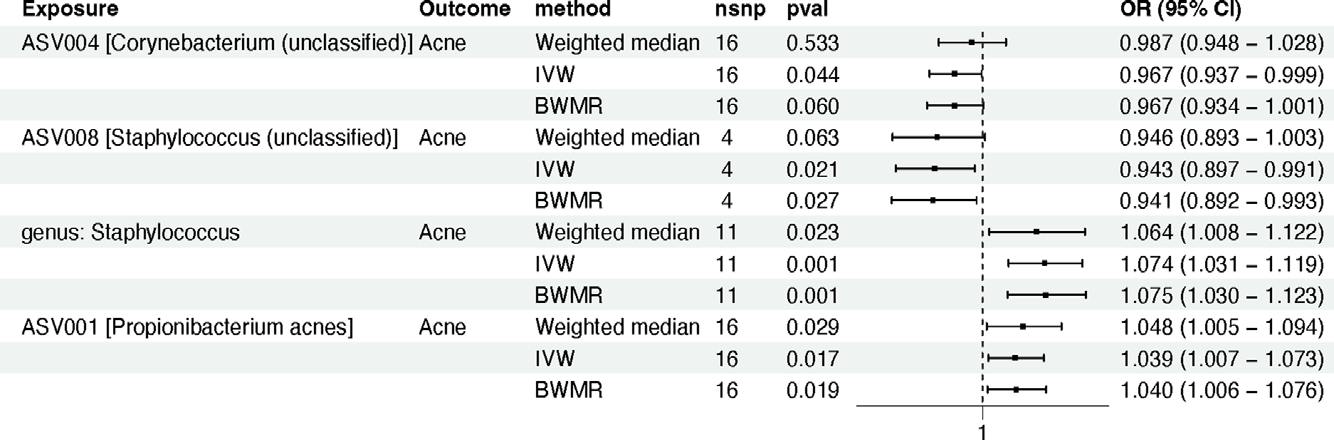
The MR analysis between 4 phenotypes of human skin microbiota and acne. ASV = amplicon sequence variant, BWMR = Bayesian weighted Mendelian randomization, CI = confidence interval, IVW = inverse-variance weighted, MR = Mendelian randomization, nSNPs = number of single nucleotide polymorphisms, OR = odds ratio.

Through systematic analysis, we identified 4 types of sebaceous skin microbiota that have a causal relationship with acne. Specifically, these are: ASV004 (Corynebacterium [unclassified]) on the forehead (IVW method: odds ratio [OR] 0.967; 95% confidence interval [CI] 0.937–0.999; *P* *=* .044), ASV008 (Staphylococcus [unclassified]) on the forehead (IVW method: OR *=* 0.943; 95% CI *=* 0.897–0.991; *P =* .021), genus Staphylococcus at the retroauricular fold (IVW method: OR *=* 1.074; 95% CI *=* 1.031–1.119; *P =* .001), and ASV001 [P. acnes] at the retroauricular fold (IVW method: OR *=* 1.039; 95% CI *=* 1.007–1.073; *P =* .017) (Fig. 2).

It is worth noting that the harmonized data for the MR analysis (Table S2, Supplemental Digital Content, https://links.lww.com/MD/P191) and the complete analysis results (Table S3, Supplemental Digital Content, https://links.lww.com/MD/P191) are available in the supplementary files. Additionally, none of the MR analyses showed evidence of heterogeneity or horizontal pleiotropy (Table S4, Supplemental Digital Content, https://links.lww.com/MD/P191). The MR-PRESSO test also did not detect any outliers. Finally, using the “leave-one-out” sensitivity analysis method, we found that systematically excluding each SNP did not substantially alter the effect estimates or the qualitative conclusions of the model (Fig. S1, Supplemental Digital Content, https://links.lww.com/MD/P192).

### 3.2. Reverse MR analysis

**Figure 3.**
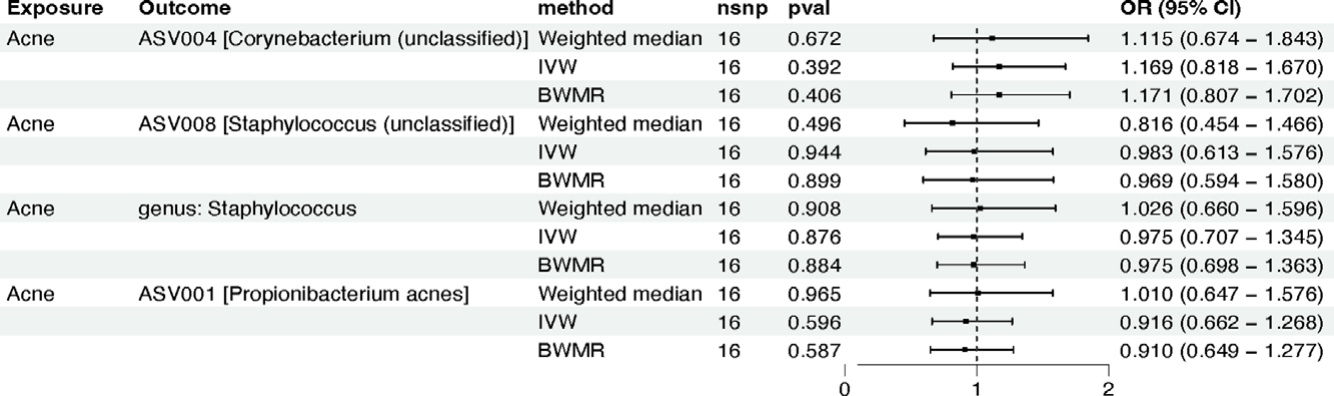
The reverse MR analysis between 4 phenotypes of human skin microbiota and acne. ASV = amplicon sequence variant, BWMR = Bayesian weighted Mendelian randomization, CI = confidence interval, IVW = inverse-variance weighted, MR = Mendelian randomization, nSNPs = number of single nucleotide polymorphisms, OR = odds ratio.

To investigate whether there is a reverse causal relationship between the aforementioned 4 types of sebaceous skin microbiota and acne, we conducted several reverse MR analyses, using these sebaceous skin microbiota as outcomes and the GWAS data for acne as the exposure. The harmonized data for the MR analyses (Table S5, Supplemental Digital Content, https://links.lww.com/MD/P191) and the complete analysis results can be found in the supplementary files (Table S6, Supplemental Digital Content, https://links.lww.com/MD/P191). Collectively, we do not find evidence of a reverse causal relationship between the aforementioned 4 types of sebaceous skin microbiota and acne (Fig. 3).

Additionally, subsequent heterogeneity testing (Table S4, Supplemental Digital Content, https://links.lww.com/MD/P191), horizontal pleiotropy testing (Table S4, Supplemental Digital Content, https://links.lww.com/MD/P191), outlier detection, and “leave-one-out” sensitivity analysis (Fig. S2, Supplemental Digital Content, https://links.lww.com/MD/P192) all yield negative results, further reflecting the robustness of our study findings.

## 4. Discussion

The human skin is inhabited by hundreds of microbial species, which occupy specific ecological niches within different skin environments, forming diverse microbial communities.^[7]^ When the balance of the normal microbiota is disturbed, or the host’s immune defense is weakened, opportunistic microbes may trigger or exacerbate skin diseases.^[8]^ The relationship between skin microbiota and acne has been long-standing, yet its complexity remains largely unexplored.^[9]^ In the skin, interactions between host and pathogen are often simplified to direct mappings between microbes and diseases, a simplification that overlooks deeper complexities.^[36]^ In this study, we conducted a bidirectional, 2-sample MR analysis using GWAS data for 23 sebaceous skin microbiota and acne, revealing a causal relationship between 4 microbes and the onset of acne. We innovatively utilized MR analysis to explore the association between multiple sebaceous skin microbiota and acne. These findings significantly contribute to our understanding of the pathogenesis of acne and offer important insights for guiding future treatment strategies.

The skin surface microbiota is primarily composed of bacteria belonging to the 3 main genera: Corynebacteria, Propionibacteria, and Staphylococci.^[37]^ The interactions among these skin microbiota members are crucial for maintaining healthy skin. The commensal bacterium P. acnes is a predominant species in sebaceous areas, playing a key role in regulating skin homeostasis and preventing colonization by other harmful pathogens,^[38,39]^ while also acting as an opportunistic pathogen in acne vulgaris. However, the causal role of P. acnes in the pathogenesis of acne is still under investigation.^[9]^ P. acnes utilizes sebum as a metabolic substrate to promote its growth and further enhances sebum secretion by increasing the activity of diacylglycerol acyltransferase, exacerbating preexisting androgen-related sebum overproduction.^[12,40]^ However, studies have found that P. acnes dominates the follicular sebaceous gland microbiota in both acne patients and healthy individuals, with similar quantities or relative abundances between the 2 groups (87%–89%),^[10]^ and even slightly higher in healthy subjects (89% vs 94%).^[11]^ This suggests that different strains of acne-associated P. acnes may play distinct roles in skin health and acne pathogenesis.^[41,42]^ Our analysis identified ASV001 (P. acnes) as a risk factor for acne onset. Therefore, our results lean towards the idea that an increase in the quantity or abundance of P. acnes is a risk factor for acne. Additionally, based on the results of reverse MR analysis, acne is not a risk factor for the proliferation of P. acnes. It is worth noting that since P. acnes is widely recognized as one of the pathogenic factors in acne, our study’s identification of the causal relationship between P. acnes and acne through MR analysis further validates the reliability of our conclusions.

Furthermore, the study found that the presence of the genus Staphylococcus in the retroauricular fold is associated with a higher risk of acne. However, ASV008 (Staphylococcus [unc.]) identified on the forehead was found to be a protective factor against acne development. This difference may stem from the varying roles and interactions of different species or subspecies of Staphylococcus in the skin microbiome. Some Staphylococcus strains may possess specific characteristics harmful to the skin, while others may have protective properties. Currently, the role of Staphylococcus as a skin commensal in acne pathogenesis remains highly debated. Some studies have identified P. acnes and Staphylococcus as the 2 most common bacteria in acne lesions,^[43]^ more prevalent in acne patients than in control groups.^[24,44]^ However, a substantial body of research has also highlighted the immunomodulatory roles of Staphylococcus, including promoting immune tolerance, controlling inflammatory responses, facilitating wound healing, and modulating immune responses.^[45]^ For instance, studies suggest that epidermal Staphylococcus can ferment glycerol to produce inhibitory zones, which help repel the overgrowth of acne-inducing P. acnes.^[18]^ Local application of short-chain fatty acids produced by glycerol fermentation of epidermal Staphylococcus significantly inhibited bacterial colonization and inflammation in acne-induced lesions in mice.^[18]^ Additionally, research by Wang et al has shown that certain strains of epidermal Staphyylococcus release succinic acid, exhibiting anti-P. acnes properties.^[46]^ Moreover, in an acne model, the application of Staphylococcus lipoteichoic acid was able to suppress inflammation by activating microRNA miR-143.^[19]^ Lastly, this study also found that ASV004 (Corynebacterium [unc.]) at the Forehead is a protective factor against acne. Currently, there is limited research on the roles of this microbiota in acne, and there is a lack of detailed information on the microbiota. Therefore, further research may be needed to differentiate between different subtypes of microbial communities in order to elucidate their roles and interactions within the skin microbiome. Such studies can help provide a more comprehensive understanding of the functions and potential impacts of specific microbial populations, offering more targeted guidance for the development of personalized skincare and treatment strategies.

Our analysis demonstrates both strengths and limitations. A significant strength lies in our focused investigation of the microbiota on the sebaceous skin surface, a common site for acne, and the exploration of its bidirectional relationship with acne. Additionally, considering that our data predominantly originates from European populations, this aspect likely reduces the bias that could arise from regional variations. However, our study is not without constraints. Firstly, our study primarily focuses on European populations, which may limit the generalizability of the findings to other ethnic groups. Genetic variations and microbiome compositions can differ significantly across populations, potentially affecting the applicability of the results. Therefore, it is necessary to conduct analyses in diverse populations to enhance the reliability and robustness of the findings. Secondly, due to the limited sample size of the skin microbiome data used for analysis, we adopted a more relaxed SNP inclusion criterion based on previous research protocols to obtain a sufficient number of SNPs for analysis. However, this approach may increase the risk of false positives, reduce the strength of IVs, and compromise the robustness of the results. Future efforts should focus on increasing the sample size to enhance the reliability of the findings. In addition, given the limitations of the sample size, only Staphylococcus was found to have a significant association with acne when using adjusted *P*-values for screening. To more comprehensively explore the potential causal relationships between skin microbiota and acne, we opted to use a threshold of *P* < .05 as the screening criterion. However, this approach may introduce multiple testing issues, increasing the risk of false-positive results. Lastly, due to data limitations, we were unable to analyze the subtypes of the microbiota, which may affect the precision of our results.

## 5. Conclusions

This study employed a bidirectional 2-sample MR analysis to explore the causal relationships between 23 sebaceous skin microbiota and the risk of acne. It was found that there are causal links between the presence of the genus Staphylococcus and P. acnes in sebaceous skin and the risk of developing acne. Furthermore, the study identified ASV004 (Corynebacterium [unclassified]) and ASV008 (Staphylococcus [unclassified]) as protective factors that could reduce the incidence of acne. This research plays an important role in exploring the relationship between skin microbiota and acne, providing significant insights into the mechanisms of acne pathogenesis and laying the groundwork for future treatments.

## Author contributions

**Conceptualization:** Yixin Xu, Haitao Wang.

**Data curation:** Jie Zhou.

**Formal analysis:** Jie Zhou.

**Funding acquisition:** Yixin Xu.

**Investigation:** Jie Zhou, Haitao Wang.

**Methodology:** Jie Zhou, Haitao Wang.

**Supervision:** Yixin Xu, Haitao Wang.

**Validation:** Haitao Wang.

**Visualization:** Yixin Xu, Haitao Wang.

**Writing – original draft:** Jie Zhou.

## Supplementary Material


