# Supplementary material for: Decoding the skin microbiota: A Mendelian randomization study reveals new insights into acne causality
Source: Medicine (Baltimore). 2025 Jun 13;104(24):e42865. doi: 10.1097/MD.0000000000042865 (PMC12173255; doi:10.1097/MD.0000000000042865)
Supplement: Supplementary file 2 [file medi-104-e42865-s002.pdf]

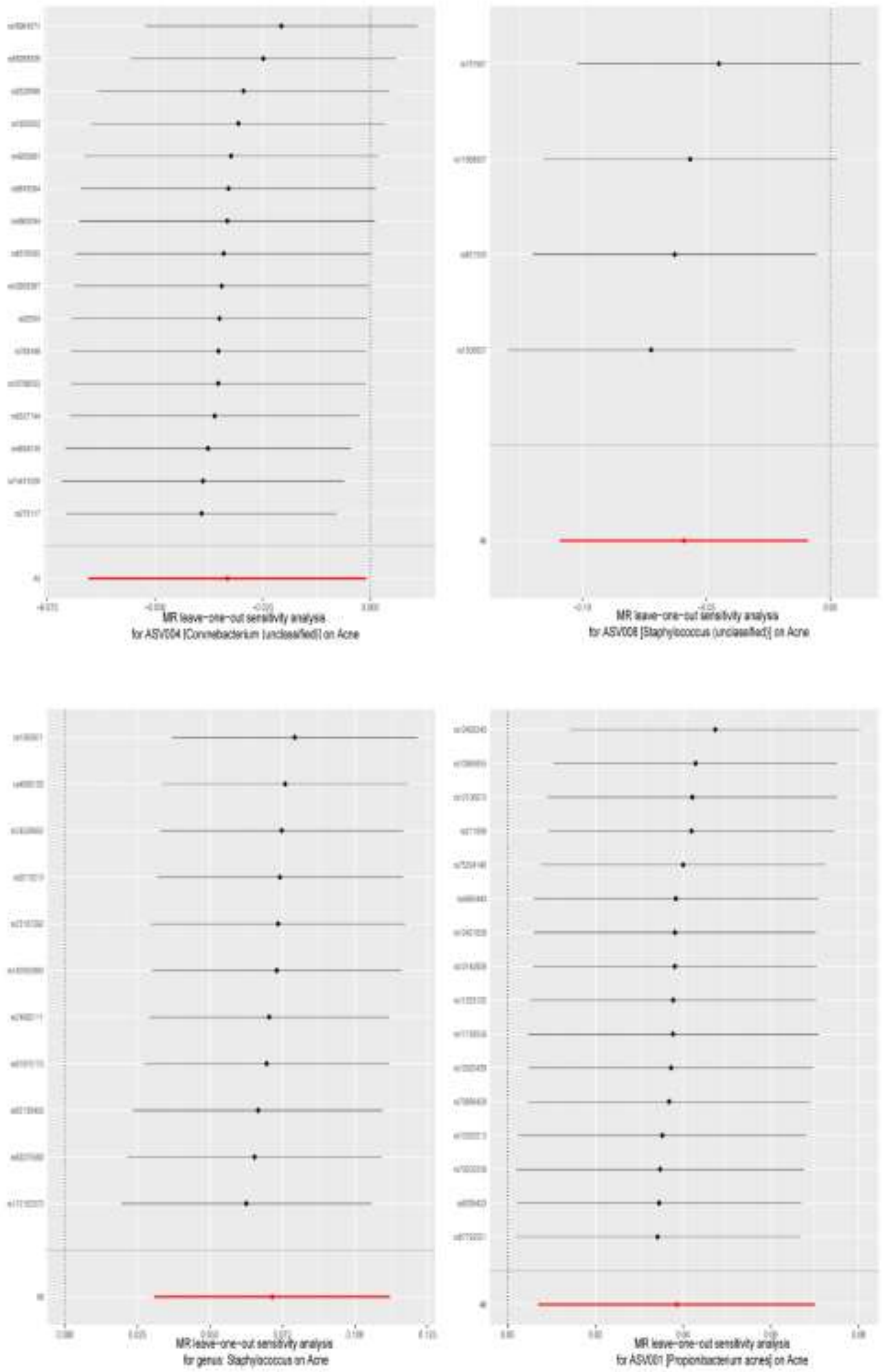

Supplementary Figure S1: Leave-one-out plot to visualize causal effect of 4 phenotypes of human skin microbiota on acne risk when leaving out one SNP. Note: SNP, single nucleotide polymorphism.

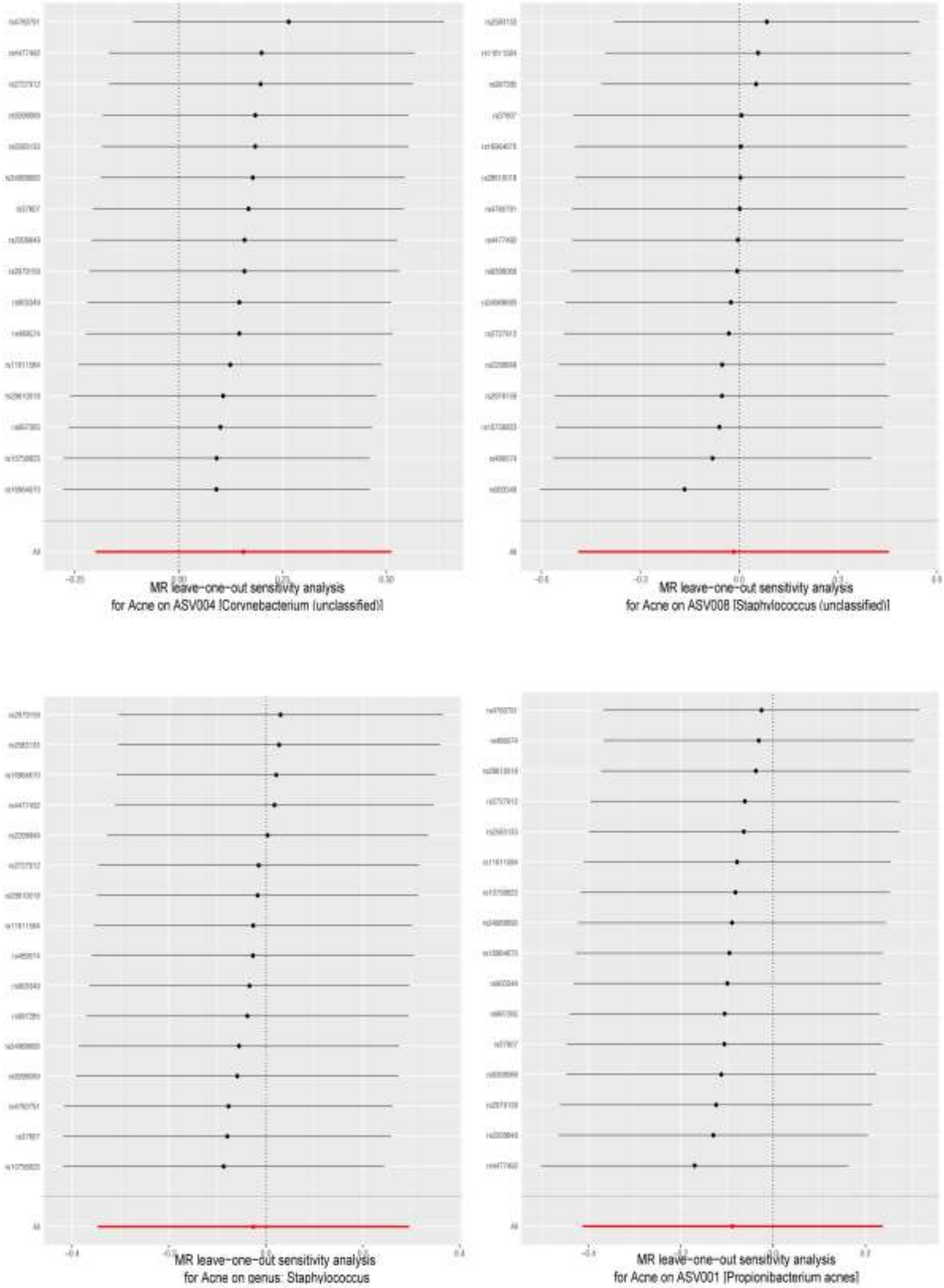

Supplementary Figure S2: Leave-one-out plot to visualize causal effect of Acne on 4 phenotypes of human skin microbiota when leaving out one SNP. Note: SNP, Single Nucleotide Polymorphism.
